# Procalcitonin expression in patients with large cell neuroendocrine carcinoma of the lung

**DOI:** 10.1186/s13104-021-05448-4

**Published:** 2021-01-15

**Authors:** Masamichi Itoga, Hisashi Tanaka, Kageaki Taima, Yoshiko Ishioka, Hiroaki Sakamoto, Shingo Takanashi, Akira Kurose, Sadatomo Tasaka

**Affiliations:** 1 Department of Respiratory Medicine, Hirosaki University Graduate School of Medicine, 5 Zaifu-cho, Hirosaki, 036-8562 Japan; 2 Hirosaki University Health Administration Center, Hirosaki, Japan; 3 Department of Anatomic Pathology, Hirosaki University Graduate School of Medicine, Hirosaki, Japan

**Keywords:** Carcinoid, Immunohistochemistry, Large cell neuroendocrine carcinoma, Procalcitonin, Pulmonary neuroendocrine tumor

## Abstract

**Objective:**

Procalcitonin (PCT) has received much attention as a serum marker for bacterial infection. Elevated serum PCT is occasionally seen in severe trauma, heatstroke, and neoplastic diseases, including lung cancer with neuroendocrine component.

**Results:**

In the present study, we evaluated PCT expression in the specimen of pulmonary neuroendocrine tumors, comparing large cell neuroendocrine carcinoma (LCNEC), carcinoid, and small cell lung carcinoma (SCLC). Pathological specimens of 10 LCNEC, 4 carcinoid, and 7 SCLC cases were evaluated with immunochemical staining of PCT. Clinical characteristics and serum levels of PCT and C-reactive protein were also evaluated. We observed positive PCT expression in 5 (50%) LCNEC and 2 (50%) carcinoid specimens that were surgically resected. Whereas serum PCT levels were not elevated in patients with PCT-positive carcinoid, two out of three LCNEC patients with high PCT expression in the tumor had elevated serum PCT levels that reflected disease progression. In patients with SCLC, PCT was not detected in the tumor or serum. This is the first immunohistochemical study of the PCT expression in the lung tumor specimens. We concluded that, in patients with LCNEC, high serum PCT levels may be indicative of disease activity and serve as a biomarker.

## Introduction

Procalcitonin (PCT) is a peptide consisting of 116 amino acids and is synthesized primarily in the C-cells of the thyroid gland as a calcitonin precursor protein [1]. In addition, inflammatory cytokines induce its production mainly in the lungs and small intestines, followed by its secretion into the blood. PCT has drawn attention as a serum marker of severe bacterial infection such as sepsis. Non-infectious PCT induction occurs when there is a major disturbance of homeostasis such as tissue injury. High levels of serum PCT are seen after surgical procedures and in serious trauma and heatstroke. Serum PCT level is known to increase in patients with medullary thyroid cancer that causes a specific production of PCT [2]. In 40.6% of cases of gastrointestinal neuroendocrine tumors (NETs), serum PCT levels are elevated and associated with NET grade and treatment effect [3].

PCT is secreted from neuroendocrine cells present in the lungs, adrenal glands, liver, kidneys, adipose tissue, and muscle during sepsis, suggesting increased production of PCT may be associated with NETs. Although elevated levels of serum PCT are occasionally seen in patients with pulmonary NETs [4], PCT expression in tumor remains to be elucidated. In this study, we evaluated PCT expression in tumor and serum PCT levels in patients with pulmonary NETs, including large-cell neuroendocrine carcinoma (LCNEC), small cell lung carcinoma (SCLC) and carcinoids.

## Main text

### Methods

#### Patient selection

We retrospectively evaluated data from 10 consecutive patients with surgically resected LCNEC and 4 with carcinoid who were diagnosed at Hirosaki University Hospital between 2013 and 2019. Pathological diagnosis of LCNEC was based upon neuroendocrine morphology and immunostaining of the neuroendocrine markers, such as chromogranin A, synaptophysin and neural cell adhesion molecule (NCAM), according to the 2015 WHO classification of lung tumors [5]. Seven patients with biopsy-proven SCLC, who underwent measurement of serum PCT at the time of diagnosis, served as controls. In total, 21 patients with pulmonary NETs were included (10 with LCNEC, 4 with carcinoid, and 7 with SCLC). None of the study subjects had fever of other signs of infection. This study was approved by the Ethics Committee of Hirosaki University Graduate School of Medicine, and written informed consent was waived because of the retrospective design.

#### Immunohistochemistry

Immunohistochemical staining was performed using anti-PCT monoclonal antibody obtained from CUSABIO (Houston, TX). Formalin-fixed and paraffin-embedded tissues were sectioned at a thickness of 4 µm and stained on positively charged glass slides stored at 4 °C within 3 days after sectioning. We used Bond-III Leica automated slide stainer, and signals were detected using the Bond polymer refine detection kit (Leica, Newcastle, UK). After peroxide blocking, staining was developed with diaminobenzidine.

#### Histological evaluation

**Fig. 1:**
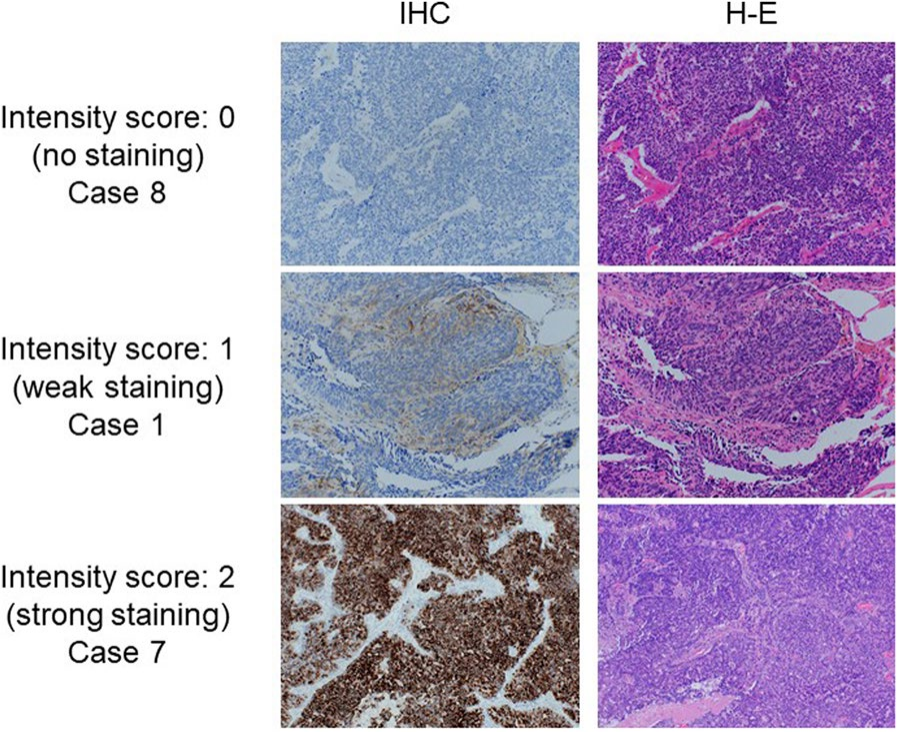
Representative images of PCT immunohistochemistry. **a** No staining (intensity score: 0) in case 8. **b** Weak staining (intensity score: 1) in case 1. **c** Strong staining (intensity score: 2) in case 7. *IHC* immunohistochemistry. *H-E* Hematoxylin–Eosin

The proportion of tumor cells found to express PCT (proportion score) was assessed according to the following scale: 0 (0–10%); 1 (11 to 25%); 2 (26 to 50%); 3 (51 to 70%); and 4 (71 to 100%) of tumor cells. The intensity of staining (intensity score) was evaluated according to the following scale: 0 (no staining); 1 (weak staining); and 2 (strong staining) in 10% of cancer cells (Fig. 1). These scores were summed to a total immunohistochemistry (IHC) score. We defined the high PCT expression as the total IHC score of 3 or higher.

#### Serum PCT assay

Serum levels of PCT were measured with luminescence immunoassay using Elecsys® BRAHMS PCT system (Roche Diagnostics, Risch-Rotkreuz, Switzerland) following the manufacturer's instructions. The lower detection limit was 0.02 ng/mL. The cut-off value was 0.5 ng/mL, which is widely used for a diagnosis of bacteremia.

### Results

#### Patient characteristics

The median age was 67 years (range, 49–70 years) in patients with LCNEC, 58 years (range, 24–68 years) in those with carcinoid, and 69 years (range, 62–78 years) in SCLC patient. There were 6 female patients (1 with LCNEC, 2 with carcinoid, and 3 with SCLC). Most of the patients with LCNEC (90%) and SCLC (86%) had smoking history. The Brinkman indices of the patients with LCNEC and those with SCLC were 653 ± 148 and 832 ± 283, respectively.

#### PCT expression in the tumors

Immunostaining for PCT was performed on 21 specimens, including 15 resected tumors and 6 transbronchial biopsy specimens (Table 1). PCT expression was observed in 5 (50%) out of 10 samples of LCNEC. Three (30%) showed high expression of PCT in the tumor, two of which revealed strong staining on 100% of tumor cells. Two (50%) of 4 carcinoid specimens demonstrated high PCT expression, one of which showed strong staining on 100% of tumor cells. None of the samples from SCLC patients showed positive staining.

**Table 1:** Procalcitonin expression and the tumor response

Case No.	Pathology	Sampling procedure	Intensity score	Proportion score	Total IHCscore	Expressionof PCT	Serum PCT (ng/mL)	Serum CRP (mg/dL)	Metastasis
1	LCNEC	Surgery	1	0	1	Low	0.05	4.17	–
2	LCNEC	Surgery	2	1	3	High	0.07	0.08	Br
3	LCNEC	Surgery	0	0	0	-	0.08	1.16	–
4	LCNEC	Surgery	0	0	0	-	N/D	< 0.02	–
5	LCNEC	Surgery	0	0	0	-	0.14	11.20	–
6	LCNEC	Surgery	0	0	0	-	0.03	0.04	–
7	LCNEC	Surgery	2	4	6	High	170.4	2.05	Br, Ad
8	LCNEC	Surgery	0	0	0	-	0.06	6.00	-
9	LCNEC	Surgery	2	4	6	High	16.72	1.90	Br, Bo, Pl
10	LCNEC	Surgery	1	0	1	Low	0.03	0.07	–
11	Carcinoid	Surgery	2	4	6	High	0.35	19.26	–
12	Carcinoid	Surgery	0	0	0	-	N/E	0.09	–
13	Carcinoid	Surgery	2	0	2	Low	0.03	< 0.02	–
14	Carcinoid	Surgery	0	0	0	-	N/E	< 0.02	–
15	SCLC	Surgery	0	0	0	-	0.07	2.80	–
16	SCLC	Bronchoscopy	0	0	0	-	0.24	2.89	–
17	SCLC	Bronchoscopy	0	0	0	-	0.03	0.06	–
18	SCLC	Bronchoscopy	0	0	0	-	0.07	3.04	–
19	SCLC	Bronchoscopy	0	0	0	-	0.04	3.05	–
20	SCLC	Bronchoscopy	0	0	0	-	0.05	0.04	–
21	SCLC	Bronchoscopy	0	0	0	-	N/D	0.09	Br

*N/D* not detected, *N/E* not examined, *Br* brain, *Ad* adrenal gland, *Bo* bone, *Pl* pleura

#### Serum PCT levels

PCT levels were measured using the serum samples collected at the time of diagnosis (Table 1). Serum PCT levels were examined in 9 out of 10 patients with LCNEC. PCT was not detected in 1 and low in 7. Two patients showed high levels of serum PCT, both of whom had intense PCT expression in the tumors. Serum PCT levels were measured in 3 out of 4 patients with carcinoid and lower than the cut-off value in all the samples examined. In 7 patients with SCLC, serum PCT was not detected in 1 and low in 6.

**Fig. 2:**
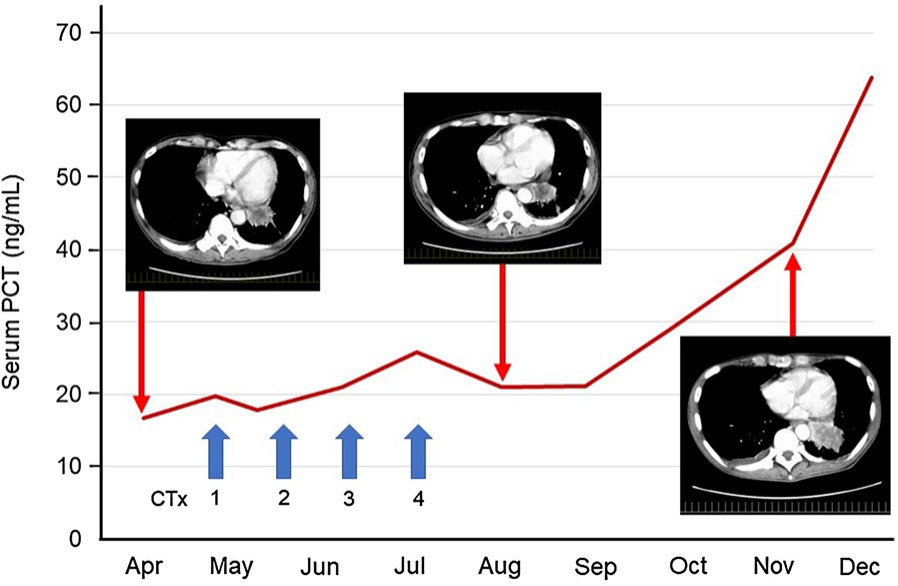
Serial changes of serum PCT levels in a patient with LCNEC. In a male patient with stage IV LCNEC, serum PCT level was around 20 ng/mL while the disease was controlled by 4 cycles of chemotherapy. Then, the PCT level was elevated as the disease progressed after the end of chemotherapy. *CTx* chemotherapy

In two LCNEC patients with elevated serum PCT levels at the time of diagnosis, we evaluated the serial changes and found that serum PCT levels changed in association with the disease progression or treatment. In Case 7, which was a stage IV disease with adrenal and brain metastases in a male patient, the disease progression could not be controlled after a total of four courses of chemotherapy. The serum PCT level was as high as 170.4 ng/mL at the time of diagnosis and gradually increased thereafter. Case 9 was also a stage IV disease with multiple brain and bone metastases and pleural dissemination in a male patient. His PCT level in serum was 16.72 ng/mL at diagnosis and around 20 ng/mL while the disease was controlled by 4 cycles of chemotherapy (Fig. 2). Then, the PCT level was elevated as the disease progressed after the end of chemotherapy.

### Discussion

PCT has been drawing attention as a serum marker of serious bacterial infection such as sepsis. In the present study, we observed positive PCT expression in a half of the specimens of surgically resected LCNECs and carcinoids. Whereas serum PCT levels were not elevated in patients with PCT-positive carcinoid, two out of three LCNEC patients with high PCT expression in the tumor had elevated serum PCT levels that reflected disease course. In patients with SCLC, PCT was not detected in the tumor or serum. To the best of our knowledge, this is the first immunohistochemical study of the PCT expression in the lung tumor specimens.

There have been some reports of elevated serum PCT in patients with primary lung cancers [4, 6, 7]. Patout and colleagues retrospectively performed a PCT dosage in the frozen serum samples of 147 patients with pulmonary neoplasia [4]. They showed that serum PCT was more elevated in those with neuroendocrine neoplasia and liver metastasis. Increased serum PCT was also associated with unfavorable prognosis [4]. Avrillon and colleagues evaluated 89 cases of newly diagnosed lung cancer with a pre-antineoplastic PCT assay and no signs of infection [6]. They found that serum PCT was positive in 42% of the cases and was strongly associated with the presence of a neuroendocrine component in histology. These findings indicated that serum PCT is occasionally elevated in patients with lung cancer, especially in those with neuroendocrine component and liver metastasis. In this study, none of the study subjects had liver metastasis, but both of 2 LCNEC patients with elevated serum PCT had remote metastases in brain and other organs. Although 5 patients with LCNEC and 2 with carcinoid had positive PCT expression in the tumor specimens, serum PCT levels were elevated only in 2 patients with LCNEC, both of whom showed high PCT expression. Taken together, at least in patients with LCNEC, elevated PCT levels in serum might be associated with high PCT expression in the tumor as well as remote metastasis.

In this study, we observed positive PCT expression in a half of the specimens of LCNEC and carcinoid, whereas none of the SCLC specimens showed PCT expression. Because all the SCLC specimens showed positive immunostaining of synaptophysin and NCAM, we considered that positive immunostaining of PCT in tissue specimens may be useful to exclude SCLC from other pulmonary NETs.

PCT is secreted from neuroendocrine cells in various organs, suggesting PCT production in NETs. In fact, immunohistochemical diagnosis of PCT-secreting NETs has been made in cases of medullary thyroid carcinoma [2] and pancreatic and gastrointestinal NETs [3, 8]. This is the first report confirming the expression of PCT in pulmonary neuroendocrine tumors.

PCT has a variety of biological effects. For example, PCT promotes the induction of nitric oxide synthase from sites of inflammation resulting in the onset of vasodilatation [9]. It also regulates cytokine reactions, inducing the onset of CD11b expression of monocytes and neutrophils [10]. These suggest that a PCT-producing cancer is progressive with poor prognosis. In fact, we observed that 2 patients with elevated PCT levels in serum had progressive disease with multiple metastasis.

### Conclusions

In this study, our immunohistochemical analysis revealed positive PCT expression in a half of the specimens of surgically resected LCNECs and carcinoids. Some of the LCNEC patients with high PCT expression in the tumor showed elevated PCT levels in serum, which was associated with disease progression. In patients with LCNEC, high serum PCT levels may be indicative of disease activity and serve as a biomarker.

## Limitations

This study has some limitations. First, the sample size was relatively small, because LCNEC is a rare type of lung cancer and its diagnosis usually needs a surgical specimen. Second, most specimens of SCLC were obtained with transbronchial biopsy, which might affect the immunohistochemical evaluation.

## Data Availability

The datasets used and analyzed during the current study are available from the corresponding author on reasonable request.

## Abbreviations

IHC: Immunohistochemistry
LCNEC: Large cell neuroendocrine carcinoma
NCAM: Neural cell adhesion molecule
NET: Neuroendocrine tumor
PCT: Procalcitonin
SCLC: Small cell carcinoma

## References

[1] Becker KL, Nylén ES, White JC, Müller B, Snider RH (2004). Procalcitonin and the calcitonin gene family of peptides in inflammation, infection, and sepsis: a journey from calcitonin back to its precursors. J Clin Endocrinol Metab.

[2] Algeciras-Schimnich A, Preissner CM, Theobald JP, Finseth MS, Grebe SKG (2009). Procalcitonin: a marker for the diagnosis and follow-up of patients with medullary thyroid carcinoma. J Clin Endocrinol Metab.

[3] Chen L, Zhang Y, Lin Y, Deng L, Feng S, Chen M, Chen J (2017). The role of elevated serum procalcitonin in neuroendocrine neoplasms of digestive system. Clin Biochem.

[4] Patout M, Salaun M, Brunel V, Bota S, Cauliez B, Thiberville L (2014). Diagnostic and prognostic value of serum procalcitonin concentrations in primary lung cancers. Clin Biochem.

[5] Travis WD, Brambilla E, Burke AP, Marx A, Nicholson AG (2015). WHO classification of tumours of the lung, pleura, thymus and heart.

[6] Avrillon V, Locatelli-Sanchez M, Folliet L, Carbonnaux M, Perino E, Fossard G, Desseigne M, Freymond N, Geriniere L, Perrot E, Souquet P-J, Couraud S (2015). Lung cancer may increase serum procalcitonin level. Infect Disord Drug Targets.

[7] Billy P-A, Parmeland L, Brunette S, Lecordier S, Pecquet M (2017). A major procalcitonin elevation without sepsis in a metastatic small cell lung carcinoma. Ann Biol Clin (Paris).

[8] Takahashi K, Ozawa E, Nakao K, Aoki S, Takase Y (2019). Hepatobiliary and pancreatic: a procalcitonin-secreting and calcitonin-secreting pancreatic neuroendocrine carcinoma. J Gastroenterol Hepatol.

[9] Hoffmann G, Totzke G, Seibel M, Smolny M, Wiedermann FJ, Schobersberger W (2001). In vitro modulation of inducible nitric oxide synthase gene expression and nitric oxide synthesis by procalcitonin. Crit Care Med.

[10] Schinkel C, Sendtner R, Zimmer S, Faist E (1998). Functional analysis of monocyte subsets in surgical sepsis. J Trauma.

